# MiR-384 Regulates the Th17/Treg Ratio during Experimental Autoimmune Encephalomyelitis Pathogenesis

**DOI:** 10.3389/fncel.2017.00088

**Published:** 2017-03-28

**Authors:** Xuebin Qu, Jingjing Han, Ying Zhang, Yuanyuan Wang, Jun Zhou, Hongbin Fan, Ruiqin Yao

**Affiliations:** ^1^Department of Neurobiology, Xuzhou Medical UniversityXuzhou, China; ^2^Department of Neurology, Affiliated Hospital of Xuzhou Medical UniversityXuzhou, China

**Keywords:** miR-384, experimental autoimmune encephalomyelitis, Th17/Treg, SOCS3, multiple sclerosis

## Abstract

Specific miRNAs are involved in the pathogenesis of multiple sclerosis (MS), during which IL-17-producing CD4^+^ T helper (Th17) cells accumulate in the central nervous system (CNS). In this study, we identified levels of miR-384 as significantly increased in mice with experimental autoimmune encephalomyelitis (EAE), an animal model of MS. Over-expression of miR-384 *in vivo* led to severe EAE, characterized by exacerbated demyelination, and increased inflammatory cell infiltration of the spinal cord; inhibition of miR-384 reversed these changes. Both the percentage of Th17, and ratio of Th17/regulatory T (Treg), cells were elevated in miR-384-transfected EAE mice, which was consistent with the observed upregulation of expression of IL-17 and the Th17 lineage-specific transcription factor, RORγt. Importantly, transfer of miR-384 overexpressing naïve T cells from wild-type (WT) to *Rag1*^−/−^ mice, which are deficient in functional autologous T and B cells, led to aggravated EAE pathogenesis, while an miR-384 inhibited group was protected from EAE. Moreover, miR-384 promoted differentiation of naïve T cells into Th17 cells *in vitro*. Furthermore, target prediction and dual luciferase reporter assays demonstrated that suppressor of cytokine signaling 3 (*SOCS3*), a gene encoding protein with an established role in Th17 differentiation, was a direct target of miR-384. Our results demonstrate an important role for miR-384 in regulation of the Th17/Treg ratio during the pathogenesis of EAE, indicating that this molecule may have potential as a biomarker and/or therapeutic target in MS.

## Introduction

Multiple sclerosis (MS) in humans, and its animal model, experimental autoimmune encephalomyelitis (EAE), are characterized by demyelination in the central nervous system (CNS), as a result of inappropriate inflammation and infiltration of CD4^+^ T cells (Jadidi-Niaragh and Mirshafiey, 2011; Rostami and Ciric, 2013). An imbalance between IL-17-producing CD4^+^ T helper (Th17) cells, which express the lineage-specific transcription factor, RORγt (Ciofani et al., 2012), and activated regulatory T (Treg) cells expressing Foxp3 (Gerriets et al., 2016) has a significant role in induction of inflammatory immune responses (Korn et al., 2009; Liu Y. et al., 2015; Zhu et al., 2016). This Th17/Treg imbalance is associated with various autoimmune and inflammatory diseases, including MS (Tzartos et al., 2008; Jamshidian et al., 2013, 2015; Lochner et al., 2015). An approach to suppress Th17 cell differentiation and correct Th17/Treg imbalances could be an effective treatment for chronic inflammatory diseases, such as MS.

Although many Th17-specific regulators have been investigated, the mechanisms underlying abnormal Th17 generation during MS pathogenesis remain elusive. MicroRNAs (miRNAs) are a class of endogenous non-coding RNAs, of approximately 23 nucleotides in length, that regulate gene expression at the post-transcriptional level (Krol et al., 2010) and are associated with various autoimmune diseases (Singh et al., 2013; Chen et al., 2016). Multiple miRNAs have roles in the development of EAE through targeting specific proteins in relevant Th17 cell pathways. Over-expression of miR-326, which targets the transcription factor, Ets-1, results in increased numbers of Th17 cells and severe EAE *in vivo* (Du et al., 2009). In mice with EAE, the expression of miR-155 is elevated in CD4^+^T cells, and mice lacking miR-155 develop milder EAE, and have fewer Th17 cells and less severe CNS inflammation (O’Connell et al., 2010; Murugaiyan et al., 2011). MiR-21 also enhances Th17 generation and mediates EAE development by promoting TGF-β signaling and limiting the inhibitory effects of IL-2 (Murugaiyan et al., 2015). In addition, miR-20b, miR-23b and miR-301a are closely associated with EAE pathogenesis through regulation of factors inducing Th17 generation, such as RORγt, STAT3 (Zhu et al., 2014), TAB2, TAB3, IKK-α (Zhu et al., 2012) and PIAS3 in the IL-6/23-STAT3 pathway (Mycko et al., 2012). Hence there is a clear and close relationship among miRNA expression, Th17 cell differentiation, and EAE pathogenesis, suggesting that specific miRNAs could serve as potential biomarkers or therapeutic targets in EAE and MS (Li et al., 2010; Zhu et al., 2012; Zhernakova et al., 2013).

In this study, we discovered that the expression of a specific miRNA, miR-384, is dramatically upregulated in EAE mice, suggesting a possible role for miR-384 in this disease. Recently, a role for miR-384 in tumor progression and neurotoxicity was identified; however, there are no previous reports of its effects in EAE pathogenesis. We found that over-expression of miR-384 resulted in promotion of Th17 cell differentiation *in vitro* and severe EAE *in vivo*, due to an excess of Th17 cells, leading to an imbalance in the Th17/Treg ratio. Hence, our results indicate that miR-384 is involved in the Th17-mediated pathogenesis of EAE.

## Materials and Methods

### Mice

C57BL/6 wild-type (WT) and *Rag1*^−/−^ mice were purchased from SLAC Laboratory Animal Co., Ltd. (Shanghai, China) and the Model Animal Research Center of Nanjing University (Nanjing, China), respectively. All experimental mice were maintained under specific-pathogen-free conditions in the animal facility of Xuzhou Medical University (Xuzhou, China). All experiments were performed in accordance with the guidelines set out by the US NIH regarding the care and use of animals for experimental procedures as well as in compliance with the Provisions and General Recommendations of the Chinese Experimental Animal Administration Legislation. The study had institutional approval from the Xuzhou Medical University Experimental Animal Ethics Committee.

### Induction of EAE

C57BL/6 WT mice (7–8 weeks old) were immunized subcutaneously with 200 μg myelin oligodendrocyte glycoprotein (MOG) peptide 35–55 (GL Biochem, China) in complete Freund’s adjuvant (Sigma-Aldrich, St. Louis, MO, USA) containing 4 mg/mL heat-killed *Mycobacterium tuberculosis* H37Ra (Difco, USA). Each mouse received 200 ng pertussis toxin (Sigma-Aldrich, St. Louis, MO, USA) twice, administered intraperitoneally (i.p.) on the day of immunization and 48 h later. Mice immunized without MOG were used as sham controls. Clinical assessment of EAE was performed daily after disease induction, and disease severity scored according to the following criteria (Du et al., 2009): (0) no clinical symptoms; (1) tail paralysis; (2) hind limb weakness or partial paralysis; (3) complete paralysis of two hind limbs; (4) paralysis of both forelimbs and hind limbs; and (5) moribund or dead.

### Isolation and Induction of Naïve T Cells

Splenocytes (SPs) or peripheral blood (PB) lymphocytes, isolated from 5- to 6-week-old C57BL/6 mice, were used to generate single-cell suspensions depleted of red-blood-cells. Naïve or CD4^+^ T cells were purified by magnetic cell sorting according to the manufacturer’s instructions (Miltenyi Biotec, Germany). For adoptive transfer studies, each recipient *Rag1*^−/−^ mouse was injected intravenously in tail veins with 5 × 10^6^ cells on the day before EAE induction. Successful reconstitution of cells was verified by determining GFP expression from the lentivirus vector (LV).

For Th17 differentiation, purified naïve T cells were cultured for 3 days under Th17-cell polarizing conditions: RPMI-1640 medium containing 10% fetal calf serum, 1 mM glutamine, 0.1 mM beta-mercaptoethanol, 1% nonessential amino acids (Sigma–Aldrich, St. Louis, MO, USA), 5 ng/mL IL-2 (R&D Systems, Minneapolis, MN, USA), 20 ng/mL IL-6, 5 ng/mL transforming growth factor-β, 10 ng/mL IL-23, 2 μg/mL anti-IL-4, 2 μg/mL anti-interferon-γ (BD Pharmingen, San Jose, CA, USA) and anti-CD3 and anti-CD28-coated beads (Invitrogen, Carlsbad, CA, USA).

### Lentivirus Preparation and Administration

For lentivirus-mediated over-expression of miR-384 (uguaaacaauuccuaggcaaugu), the genomic sequence spanning the mouse miR-384 coding region and ~70 bp of flanking sequence was cloned into the lentiviral vector, H1-MCS-CMV-EGFP (GV-209, Genechem, Shanghai). For lentivirus-mediated inhibition of miR-384, miR-384 complementary (acauugccuaggaauuguuuaca) or negative control (uucuccgaacgugucacgu) sequences were cloned into the same vector. Lentiviruses were produced by Shanghai Genechem Company. Target naïve T cells were infected at a multiplicity of infection of 3 for 12 h, according to the user’s manual, before induction of Th17 polarization. For administration to mice, approximately 2 × 10^7^ transforming units of recombinant lentiviruses were delivered by injection into tail veins 7 days before EAE induction. GFP^+^ T cells were collected for further examination.

### Flow Cytometric Analyses

PB-, lymph node-, or spinal cord-infiltrating lymphocytes were prepared by Percoll gradient centrifugation and then incubated with Cell Stimulation Cocktail (eBioscience, San Diego, CA, USA) for 5 h. For intracellular staining, single-cell suspensions were prepared, surface-stained with FITC-labeled anti-CD4 antibody (Miltenyi Biotec, Germany), and then fixed and permeabilized using a Fixation/Permeabilization Kit (BD Biosciences, San Jose, CA, USA). Subsequently, the cells were washed and stained with Anti-IL-17-PE antibody (Miltenyi Biotec, Germany). Co-staining with RORγt, Foxp3, suppressor of cytokine signaling 3 (SOCS3) rabbit lgGs (CST, USA) was performed for some experiments, and APC-Goat anti-Rabbit lgG (Miltenyi Biotec, Germany) were used as secondary antibodies. Analyses were performed using a MACSQuant^TM^ Flow Cytometer (Miltenyi Biotec, Germany), and the results were analyzed using FlowJo7.6. Protein expression levels are expressed as mean fluorescence intensity (MFI).

### Quantitative RT-PCR

Total RNA, from CD4^+^ T cells or spinal cords from mice anesthetized by i.p. overdose of pentobarbital (150 mg/kg), was extracted using Trizol reagent (Ambion, USA). Quantitative RT-PCR analyses of miR-384 levels were performed using SYBRGreen miRNA assays (Genechem, Shanghai, China) with U6 small nuclear RNA as an internal reference for normalization. Myelin basic protein (MBP) mRNA was examined using a SYBR Green real-time PCR kit on a LightCycler^®^ 480II System (Roche, Switzerland). Relative expression of miRNA or mRNA was evaluated using the 2^−△△ct^ method and expression levels were normalized relative to those of U6 or β-actin, respectively. The primers used were as follows: MBP, 5′-ACCCTCACAGCGATCCAAGT-3′ and 5′-TACGGCTCGGAGCTCACC-3′; and β-actin, 5′-GAGACCTTCAACACCCCAGCC-3′ and 5′-AATGTCACGCACGATTTCCC-3′.

### Histological Analyses

For histological analyses, mice were anesthetized with pentobarbital, and then perfused through the left ventricle, first with normal saline to eliminate the blood and then with buffered 4% paraformaldehyde. Spinal cords were embedded in paraffin, and 4 μm thick sections that had been deparaffinized and rehydrated were stained with hematoxylin and eosin (H&E) or luxol fast blue (LFB) for analysis of inflammation and demyelination. Demyelination was quantified in LFB stained sections by calculation of the area of demyelination relative to the total area analyzed. Spinal cord infiltrates were quantified as infiltrates per mm^2^ in H&E stained sections.

For transmission electron microscopy analyses, fixed and embedded samples were cut into ultra-thin sections (80 nm) and stained with uranyl acetate and lead citrate. Electron micrographs were captured using an FEI Tecnai G2 T12 transmission electron microscope (USA). The axonal diameter (d) was defined as the shortest distance across the center of the axon, omitting the myelin sheath thickness. Fiber diameter (D) was defined as the axonal diameter plus the total myelin sheath thickness on both sides. The g-ratio was calculated as d/D.

For immunofluorescence analyses, prepared spinal cords were incubated overnight in sodium phosphate buffer containing 30% sucrose, then embedded in Optimal Cutting Temperature medium (Leica, Germany) for sectioning. Cryosections (15 μm) were thawed and washed with 0.01% PBS with 5% BSA and 0.3% Triton X-100, and then incubated with MBP monoclonal antibody (CST, USA) for 1 h at 37°C. Following an additional wash step, specimens were incubated with FITC-Goat IgG (LI-COR, USA) and the cell nuclei counterstained with 4′,6-diamidino-2-phenylindole (DAPI). Images were acquired using a fluorescence microscope system (Olympus, Japan) and MFI values calculated using ImageJ software.

### ELISA

Cerebrospinal fluid was collected from LV-transfected mice according to a previously described protocol (Liu and Duff, 2008). An IL-17 ELISA kit was purchased from Westang (Shanghai, China), and ELISA performed according to the manufacturer’s protocol.

### Western Blotting

The spinal cord was stripped and then ultrasonically homogenized in RIPA buffer. Protein concentrations were measured using a bicinchoninic acid protein assay kit (Beyotime, Shanghai, China). Protein samples were separated by electrophoresis in SDS denaturing 10% polyacrylamide gels (Beyotime, Shanghai, China) and transferred to nitrocellulose membranes, which were subsequently blocked in 0.01% PBS containing 5% BSA. Then membranes were incubated with MBP and β-actin monoclonal antibodies (CST, USA). After washing, the membranes were incubated with appropriate IRDye-conjugated secondary antibodies (LI-COR, Lincoln, NE, USA), scanned using an Odyssey Infrared Imaging System Scanner (LI-COR, Lincoln, NE, USA), and the results analyzed using ImageJ software.

### Dual Luciferase Reporter Assay

A fragment of the 3′-UTR of *SOCS3*, containing the WT predicted miR-384 binding site, or a mutated form of the site, and flanking sequences containing suitable enzyme cleavage sites, was synthesized by Sangon company (Shanghai, China) and cloned downstream of the luciferase reporter gene. The luciferase reporter vectors, together with plasmids expressing Renilla luciferase, and miR-384 or control miR sequences, were each transfected into 293T cells using Lipofectamine 2000 reagent (Invitrogen, Carlsbad, CA, USA), following the manufacturer’s instructions. Cells were harvested 24 h after transfection and assayed for luciferase activity using a Dual-Luciferase Reporter Assay System (Promega, Madison, WI, USA).

### Statistical Analyses

Two-tailed Student’s *t* tests, two-way analysis of variance followed by Bonferroni’s *post hoc* test for multiple comparisons, and Mann-Whitney tests (for nonparametric data; EAE score) were used to evaluate differences between groups. The results are expressed as means ± standard deviation (SD). Differences were considered statistically significant when *p* < 0.05, and statistically significant data are indicated by asterisks (*p < 0.05, **p < 0.01).

## Results

### miR-384 Regulates EAE Pathogenesis

**Figure 1:**
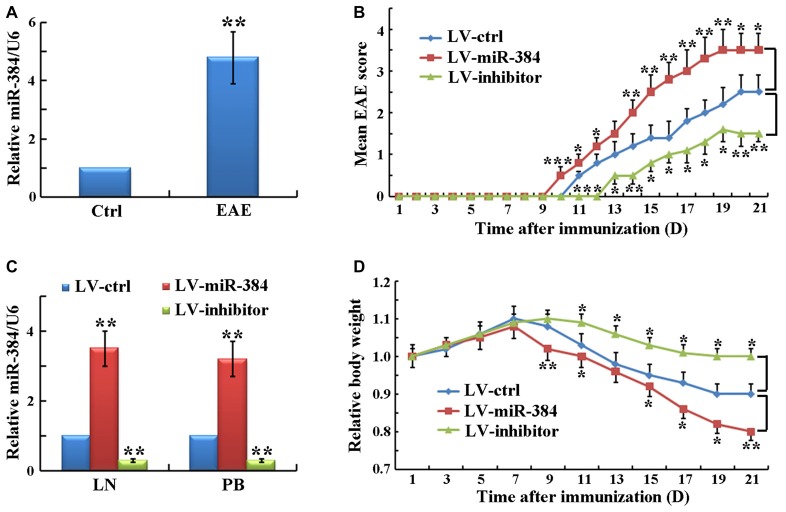
**MiR-384 regulates encephalomyelitis (EAE) pathogenesis. (A)** Quantitative RT-PCR analyses of miR-384 expression in peripheral blood (PB) naïve CD4^+^ T cells sorted from control (ctrl) and EAE mice on day 21 after immunization (*n* = 3 mice per group). Relative miRNA expression levels were evaluated by the 2^−△△ct^ method and normalized to the expression of U6. The control was set as 1.** (B)** Mean EAE scores of lentivirus vector (LV)-transfected mice were recorded daily (*n* = 5 mice per group).** (C)** Quantitative RT-PCR analyses of miR-384 levels in CD4^+^ T cells from lymph nodes (LN) and PB of LV-transfected mice 7 days later (*n* = 3 mice per group). **(D)** Body weights of LV-transfected mice normalized to the initial body weight of each mouse (*n* = 5 mice per group). Data are presented as means ± standard deviation (SD). *p < 0.05, **p < 0.01 and ***p < 0.001. Data shown are single representative results from three independent experiments. LV-ctrl, control miR; LV-miR-384, miR-384 sequence; and LV-inhibitor, miR-384-inhibitor.

To identify specific miRs involved in EAE development, we determined the expression levels of miRs in PB CD4^+^ T cells, which have key roles in EAE pathogenesis. The results demonstrated that the expression of miR-384 was significantly elevated in EAE relative to control mice (Figure 1A). To study the function of miR-384, we injected lentiviral particles expressing the miR-384 sequence (LV-miR-384), an miR-384 inhibitor (LV-inhibitor), or control miR (LV-ctrl) into mice before EAE induction. There were no statistically significant differences in EAE induction in non-infected, empty-vector-infected and LV-ctrl-infected EAE mice (data not shown), indicating that the vector and control sequences had no effect on EAE. Over the 21 days after immunization, clinical assessment of EAE demonstrated that LV-miR-384 mice had earlier onset (day 10) and higher mean disease severity scores (maximum score of 4.0 on day 19) than controls, in which mice began to suffer from EAE on day 11 and the maximum score was 3.0 on day 20. Moreover, deficiency of miR-384 caused by LV-inhibitor led to mild EAE from day 13, with a maximum score of only 1.5, on day 19 (Figure 1B). In the lymph nodes (LN) and PB lymphocytes of mice injected with LV-miR-384, the expression of miR-384 was more than three times that in controls, while levels in LV-inhibitor mice were one-third of those in controls (Figure 1C). Moreover, LV-miR-384 mice suffering from EAE exhibited the greatest weight loss, while LV-inhibitor group mice lost the least weight (Figure 1D). These results imply a potential initiating role for miR-384 in the development of EAE.

### miR-384 Enhances Demyelination in the Spinal Cord during EAE

**Figure 2:**
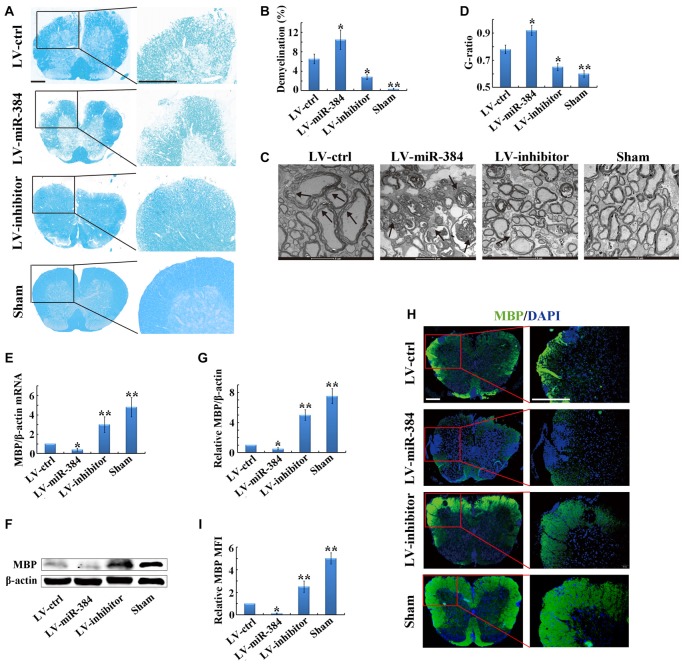
**MiR-384 enhances demyelination in the spinal cord during EAE.** EAE mice were sacrificed on day 21 after immunization for the following analyses. Sham controls, mice immunized without myelin oligodendrocyte glycoprotein (MOG) peptide. **(A)** Representative luxol fast blue (LFB) staining of spinal cords. Boxed areas in left images are enlarged in the images on the right. Scale bars, 50 μm. **(B)** Quantification of demyelination in **(A)**. **(C)** Transmission electron micrographs of spinal cords. Arrows show myelin sheath damage. Scale bars, 2 μm. **(D)** Analysis of g-ratios in **(C)**. **(E)** Quantitative PCR analyses of Myelin basic protein (MBP) mRNA expression in spinal cords (*n* = 3 mice per group). Relative expression of mRNA was evaluated by the 2^−△△ct^ method and normalized to the expression of β-actin. The control was set as 1. **(F,G)** Western blotting analyses of MBP in spinal cords (*n* = 3 mice per group). **(H)** MBP immunofluorescence staining of spinal cords. Boxed areas in left images are enlarged in right images. Scale bars, 50 μm. **(I)** Statistical analyses of mean fluorescence intensity (MFI) of MBP in H (*n* = 5 per group). Data are presented as means ± SD. *p < 0.05 and **p < 0.01. Data shown are single representative results from three independent experiments.

To further clarify the impact of miR-384 on the pathogenesis of EAE, we performed histological analyses of spinal cord sections by LFB staining. The results indicated that LV-miR-384 mice had the largest areas of demyelination, while the spinal cords of LV-inhibitor mice were relatively intact (Figures 2A,B). Observation by transmission electron microscopy revealed very loose and disintegrated myelin sheaths in the spinal cords of LV-inhibitor and LV-ctrl EAE mice (Figures 2C,D). Next, we examined the expression of MBP, which is indispensable for the process of myelination, and found that it was downregulated in LV-miR-384 mice, and upregulated in the LV-inhibitor group (Figures 2E–G). Similarly, LV-miR-384 mice had severe myelin sheath damage in the spinal cord, with the largest area of MBP loss (Figures 2H,I). Taken together, these findings indicate that miR-384 exacerbates demyelination in the spinal cord during EAE pathogenesis.

### miR-384 Disturbs the Th17/Treg Ratio during EAE

**Figure 3:**
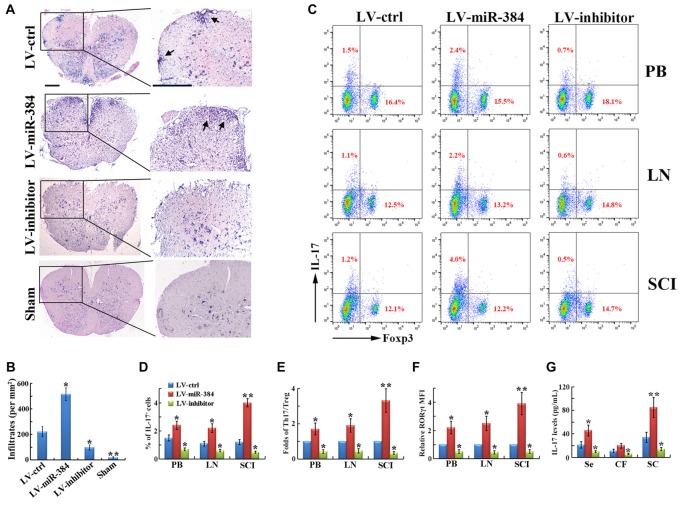
**MiR-384 disrupts the Th17/Treg ratio during EAE. (A)** Representative hematoxylin and eosin (H&E) staining of spinal cord sections from LV-transfected and sham control mice. Boxed areas in left images are enlarged in right images. Arrows show lymphocyte infiltration. Scale bars, 50 μm. **(B)** Quantification of spinal cord infiltrates in **(A)**. **(C)** Representative flow cytometric analyses of IL-17^+^ and Foxp3^+^ cells among CD4^+^ gated cells from stimulated PB, LN and spinal cord infiltrating (SCI) lymphocytes. **(D,E)** Statistical analyses of the percentage of IL-17^+^ cells and the ratio between IL-17^+^ Th17 and Foxp3^+^ Treg cells in (**B**; *n* = 3 per group). **(F)** MFI analyses of flow cytometry for RORγt (*n* = 3 per group). **(G)** The concentration of IL-17 in serum, cerebrospinal fluid and spinal cord homogenate was measured by ELISA (*n* = 5 per group). Data are presented as means ± SD. *p < 0.05 and **p < 0.01. Data shown are single representative results from three independent experiments.

EAE is an autoimmune disease model characterized by severe CNS inflammation. In our study, LV-miR-384 mice developed more extensive inflammatory cell infiltration in the spinal cord (Figures 3A,B), consistent with the observed increases in demyelination and MBP loss (Figure 2). In flow cytometric analyses, we found that the percentage of IL-17^+^ Th17 cells among PB, lymph node and spinal cord infiltrating (SCI) lymphocytes increased more in LV-miR-384 mice than in LV-ctrl mice (Figures 3C,D), while the percentage of Foxp3^+^ Treg cells showed no obvious differences among the three groups (Figure 3C). Thus, the relative ratio of Th17 to Treg cells was largest in LV-miR-384 mice (Figure 3E). Moreover, the increased number of Th17 cells in LV-miR-384 mice was confirmed by the observation that the expression of RORγt was upregulated (Figure 3F), along with IL-17 levels (Figure 3G). Therefore, miR-384 appears to impact Th17/Treg ratios in EAE by increasing the number of Th17 cells.

### miR-384 Promotes the Generation of Th17 Cells

**Figure 4:**
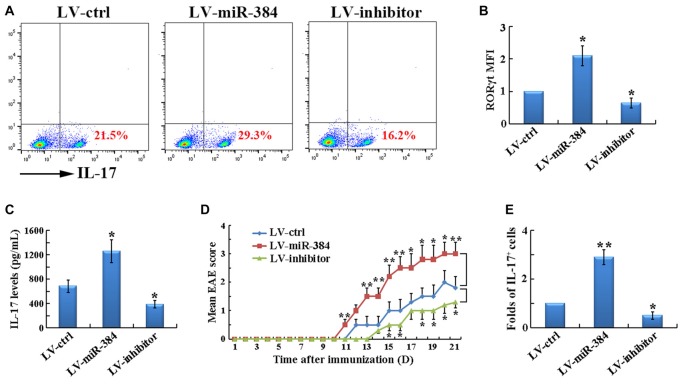
**MiR-384 promotes the generation of Th17 cells.** For the following analyses, naïve T cells were transfected for 12 h and GFP^+^ cells collected for tests. **(A)** Flow cytometric analyses of stimulated IL-17^+^ cells among the CD4^+^ gated cells after 3 days of induction under Th17-polarizing conditions. MFI analyses of **(B)** flow cytometry for RORγt and **(C)** ELISA of IL-17 in culture supernatants were also performed (*n* = 3 per group). **(D)** Mean EAE scores of *Rag1*^−/−^ mice transfected with naïve T cells (*n* = 5 mice per group). **(E)** Relative ratio of Th17 cells in SCI lymphocytes from the mice in **(D)** on day 21 after immunization, determined by flow cytometric analyses (*n* = 3 per group). Data are presented as means ± SD. *p < 0.05 and **p < 0.01. Data shown are single representative results from three independent experiments.

To determine the role of miR-384 in Th17 differentiation, naïve T cells were transfected with LV-miR-384, LV-inhibitor, or LV-ctrl and then cultured under Th17-polarizing conditions. Over-expression of miR-384 resulted in a higher percentage of IL-17^+^ cells (Figure 4A). Accordingly, the expression of RORγt (Figure 4B) and secretion of IL-17 (Figure 4C) were also significantly upregulated by miR-384. Importantly, transfer of the transfected naïve T cells into age-matched *Rag1*^−/−^ mice, which are deficient in functional T and B cells and hence protected from EAE, restored the susceptibility of these mice to EAE development; furthermore, mice that received LV-miR-384-transfected cells suffered from the most severe EAE, characterized by the earliest onset (on day 11), highest disease severity score (approximately 3.5; Figure 4D), and the greatest number of IL-17^+^ Th17 cells in spinal cord infiltrates (Figure 4E). Collectively, these data demonstrate that miR-384 promotes the generation of Th17 cells.

### miR-384 Directly Targets *SOCS3*

**Figure 5:**
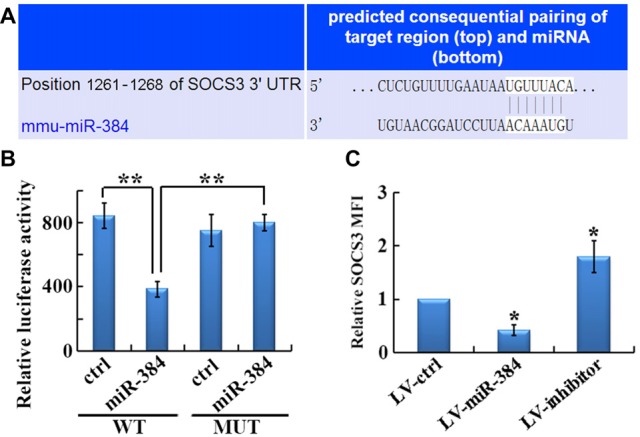
***SOCS3* is a direct target of miR-384. (A)** Illustration of the sequence match between miR-384 and *SOCS3* mRNA determined using TargetScan. **(B)** Luciferase activity of reporter vectors containing wild-type (WT) or mutated (MUT) *SOCS3* 3′-UTR co-transfected with miR-384 or control miR (ctrl; *n* = 3 per group). **(C)** MFI analyses of flow cytometry for SOCS3 in GFP^+^ naïve T cells 3 days post-transfection (*n* = 3 per group). Data are presented as means ± SD. *p < 0.05 and **p < 0.01. Data shown are single representative results from three independent experiments.

The identification of gene targets for individual miRs is key to understanding the molecular mechanisms underlying miR-mediated regulation. Among candidates predicted by TargetScan, we identified *SOCS3*, a gene encoding a protein with an established role in Th17 differentiation, whose 3′-UTR mRNA sequence closely matched the miR-384 target site (Figure 5A). We then conducted luciferase reporter assays, which demonstrated that luciferase activity was reduced by 50% in cells co-transfected with the *SOCS3* WT luciferase reporter and miR-384, compared with miR-ctrl (Figure 5B, WT). However, site-directed mutagenesis of the miR-384 recognition site in the *SOCS3* 3′-UTR abolished the inhibitory effects of miR-384 (Figure 5B, MUT). Consistently, the expression of *SOCS3* in naïve T cells over-expressing miR-384 was clearly lower than that in controls, while downregulation of miR-384 led to increased *SOCS3* expression levels (Figure 5C). Taken together, these results show that miR-384 can regulate *SOCS3* expression through a partially complementary binding site in the 3′-UTR.

## Discussion

An appropriate balance between the pro-inflammatory Th17 cell population and immunosuppressive Treg cells is critical for immune homeostasis (Wu et al., 2016); imbalances are associated with various inflammatory and autoimmune diseases, including MS, which is linked to a shift towards increased Th17 cell levels (Venkatesha et al., 2015). Here, we report that miR-384 over-expression leads to an imbalance in Th17/Treg ratios, resulting in exacerbation of EAE with severe demyelination, through the promotion of Th17 cell differentiation by targeting *SOCS3*. This miR-384-associated Th17/Treg imbalance represents a potential therapeutic target for MS and a novel indicator of disease risk (Yu et al., 2015).

The SOCS family consists of intracellular proteins that are key physiological regulators of the immune system. SOCS3 is an important member of the SOCS family and can negatively regulate the IL-6-JAK-STAT3 pathway, which is indispensable for Th17 cell differentiation (Qu et al., 2012; Liu X. et al., 2015). SOCS3-deficient T cells show hyperactivation of STAT3 and significantly increased secretion of IL-17, induced by IL-6 (Chen et al., 2006). Therefore, miR-384 promotes the differentiation of Th17 cells, which disturbs the Th17/Treg ratio, through targeting of *SOCS3*. However, there do not appear to be any direct effects of miR-384 on Treg cell development. This may be because SOCS3 is involved in specific inhibition of STAT3, but not STAT5, signaling. The STAT5 pathway is activated by TGF-β to induce Foxp3 expression and Treg cell development (Basu et al., 2015). The mutually inhibitory roles of Th17 and Treg cells may be responsible for the slight decrease in Treg cells when Th17 cell generation is promoted by miR-384 in EAE mice and their slight increase when miR-384 is knocked down (Figure 3C).

miR-384, the role of which has previously been poorly understood, has recently been the focus of research attention. miR-384 has suppressive effects on tumor cell proliferation through the direct inhibition of *IRS1* expression (Lai et al., 2016), and contributes to aberrant Runx expression in prostate tumors (Farina et al., 2016). In addition, several studies have demonstrated a relationship between miR-384 and the CNS. Gu et al. (2015) used next-generation sequencing to profile miRNA transcriptomes, and found that miR-384 is required for enduring synaptic and spinal plasticity by regulation of the expression of *RSK3*. Another study demonstrated that miR-384 is a promising candidate as a biomarker of spinal cord injury, because of a significant increase in miR-384 in the serum, which correlates with injury severity (Hachisuka et al., 2014). In addition, miR-384 is elevated in the hippocampus during neural cell death and is, therefore, considered to have potentially novel roles in neurotoxicity (Ogata et al., 2015) and the development of Parkinson’s disease (Jiang et al., 2016) and Alzheimer’s disease (Liu et al., 2014). In this study, we discovered another novel function of miR-384, namely a critical involvement in the development of the Th17/Treg imbalance and demyelination in the CNS during the pathogenesis of EAE through targeting of *SOCS3*.

The fact that an increase of the Th17/Treg ratio in MS patients vs. healthy controls (Jamshidian et al., 2013) implies recovering the balance between Th17 and Treg cells may be an important target in the context of inflammation. All trans retinoic acid (ATRA), as a RORγt antagonist, has been proved to ameliorate EAE severity via the following mechanisms: (1) inhibition of Th17 cell differentiation by binding to the RARα sequence to downregulate RORγt expression and then increase Foxp3^+^ T cells (Hall et al., 2011; Egan et al., 2016); and (2) Th17 cell dysfunction due to the decrease in IL-23 and IL-6 receptor expression accompanied by the promotion of TGF-β signaling through the Smad3 pathway in Treg cells (Klemann et al., 2009; Abdolahi et al., 2015). Recent transcriptional network analysis has identified a core set of transcription factors, such as STAT3, which are crucial for the differentiation of Th17 cell by inducing RORγt expression and DNA binding (Ciofani et al., 2012). In addition, distinct miRNAs, the post-transcriptional modulators of RNA stability, have been reported to regulate the Th17 cell differentiation by modulating the amount of transcripts that encode crucial positive regulators of Th17 cell development (Korn and Kallies, 2017). It is encouraging that the miR-based therapy has been in clinical trials (Gebert et al., 2014), offering a novel therapeutic approach if miRNAs can be effectively applied *in vivo*.

In conclusion, we found that the expression of miR-384 is greatly increased during the pathogenesis of EAE, and that its over-expression *in vivo* leads to severe EAE, due to aggravated demyelination caused by the promotion of Th17 cell generation. In addition, this microRNA promotes Th17 cell differentiation through targeting *SOCS3*. These results facilitate better understanding of the pathogenesis of EAE and imply that miR-384 may be an effective biomarker or potential therapeutic target in MS.

## Author Contributions

XQ and RY designed the study, performed experiments, analyzed the data and wrote the article; JH, YZ, YW and JZ performed experiments; HF and RY reviewed the manuscript. All authors read and edited the manuscript.

## Funding

This study was supported by grants from the National Natural Science Foundation of China (No.81271345 to RY, 81302519 to XQ), the Natural Science Foundation of Jiangsu Province (No. BK20131132 to RY, BK20130221 to XQ), 333 Project (XQ) and Qing Lan Project (RY) of Jingsu Province, College Graduate Research and Innovation Project of Jingsu Province (KYLX16_1131 to YZ), and Xuzhou Medical University Scientific Research Fund for Talents (2015KJ07 to JH).

## Conflict of Interest Statement

The authors declare that the research was conducted in the absence of any commercial or financial relationships that could be construed as a potential conflict of interest.
